# Accuracy Enhancement for Forecasting Water Levels of Reservoirs and River Streams Using a Multiple-Input-Pattern Fuzzification Approach

**DOI:** 10.1155/2014/432976

**Published:** 2014-03-24

**Authors:** Nariman Valizadeh, Ahmed El-Shafie, Majid Mirzaei, Hadi Galavi, Muhammad Mukhlisin, Othman Jaafar

**Affiliations:** ^1^Department of Civil and Structural Engineering, Universiti Kebangsaan Malaysia (UKM), 43000 Bangi, Selangor, Malaysia; ^2^Civil Engineering Department, Faculty of Engineering, Universiti Putra Malaysia (UPM), 43300 Serdang, Selangor, Malaysia

## Abstract

Water level forecasting is an essential topic in water management affecting reservoir operations and decision making. Recently, modern methods utilizing artificial intelligence, fuzzy logic, and combinations of these techniques have been used in hydrological applications because of their considerable ability to map an input-output pattern without requiring prior knowledge of the criteria influencing the forecasting procedure. The artificial neurofuzzy interface system (ANFIS) is one of the most accurate models used in water resource management. Because the membership functions (MFs) possess the characteristics of smoothness and mathematical components, each set of input data is able to yield the best result using a certain type of MF in the ANFIS models. The objective of this study is to define the different ANFIS model by applying different types of MFs for each type of input to forecast the water level in two case studies, the Klang Gates Dam and Rantau Panjang station on the Johor river in Malaysia, to compare the traditional ANFIS model with the new introduced one in two different situations, reservoir and stream, showing the new approach outweigh rather than the traditional one in both case studies. This objective is accomplished by evaluating the model fitness and performance in daily forecasting.

## 1. Introduction

Reservoirs and river basins are the most significant components of water resource management, providing effective multipurpose water storage that is employed for irrigation, water supply, hydropower, and flood drought control; to most effectively use this stored water, it is essential to optimally monitor the reservoir level to obtain the desired performance. The lack of future information regarding the inflow, water storage, and parameters that influence the reservoir level (e.g., amount of rainfall, water release, evaporation, soil moisture, geomorphology of the watershed, and infiltration) represents uncertainties that must be considered in water resource operation. In river flow studies, forecasting is normally forecasting of either water level [1–4] or runoff [5–9].

In primary attempts, the prediction of reservoir levels relied on linear mathematical relationships determined on the basis of the experience of the operators, mathematical curves, and guidelines [10]. In recent decades, the artificial neural network (ANN) method was the first method of artificial intelligence introduced to water resource management providing better performance in the modeling of the nonlinear systems and making predictions than traditional models, such as moving average methods. These new approaches were applied to rainfall-runoff subsets due to a large number of uncertainties in the watershed parameters, hydrological aspects, and operational decisions [11–14]. Recently, various combinations of ANN and other methods, including fuzzy sets (FS) [15, 16], genetic algorithm programming (GP) [17, 18], support vector machine (SVM) [19, 20], and types of swarm intelligence [21], have introduced new modeling systems with different strengths that are able to address the weaknesses of previous methods.

The neurofuzzy modeling system has become a widely accepted method due to the If-Then rules' flexibility and ability to learn from empirical data [22]. Moreover, a major advantage of the ANFIS model is dividing the input space into fuzzy subspaces and mapping the output using a set of linear functions. Because nonlinear relationships and a large number of data sets existed in hydrological studies, the new time series methods have become more preferable. The adaptive network based fuzzy interface system (ANFIS) was introduced in water resource by L.-C. Chang and F.-J. Chang in 2001 [23]. After that, various types of hydrological modeling, water quality assessment, groundwater issues, and water resource uncertainties have been modeled by ANFIS [16, 24–27].

It is noticed that, according to the previous studies done in estimation the water level in reservoirs and streams, the ANFIS model provides better accuracy rather than the other conventional and data-driven methods [28, 29].

In river flow studies, forecasting is divided to forecasting the water level [1–4, 30] or the amount of discharge the water [5–9].

Past studies that have used ANFIS for water resource applications have not taken into account the input criteria and they have only used one type of membership function to “fuzzify” the various inputs in the modeling system. The objective of this study is to evaluate the estimation of water level in reservoirs and river basins utilizing two case studies, the Klang Gates Dam (Figure 5) and Rantau Panjang station on the stream of Kota Tinggi in Malaysia, using different types of membership functions for fuzzifying each type of crisp inputs in the adaptive neurofuzzy interface modeling system to show the influence of considering the specific MF for each crisp input on the basis of the input criteria in water resource area.

The data source for training the model can be from same or different type of data. The data types that are used as data source for data training are river flow [2, 5, 7, 31], rainfall [8, 9, 32], water level and rainfall [1, 3, 33], water level and sea level pressure [4], and flow, rainfall, temperature, and snowmelt [34, 35].

The following study describes the structure of the ANFIS model used to forecast the future level of water based on the historical rainfall and water level data in two different models for each type of MF. Next, the results and statistical criteria were examined to compare the models' accuracies. Finally, the conclusion of the study evaluates the accurate model, the Klang Gates Dam and Rantau Panjang station on the stream of Kota Tinggi in Malaysia.

## 2. Methodology

**Figure 1:**
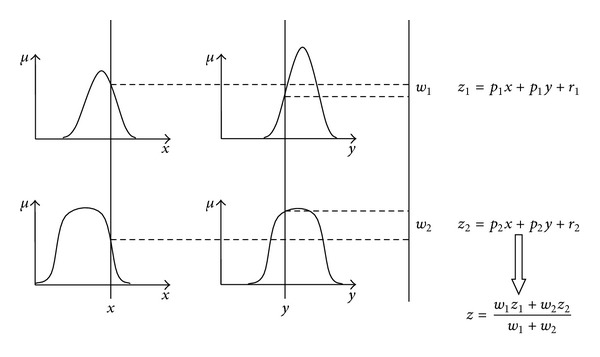
The Sugeno-type fuzzy model.

In this study a distinct type of membership function considers for each type of input on the basis of the input criteria in the ANFIS architecture determining the efficiency of the new method on forecasting the water level of river basins and reservoirs. Fundamentally, ANFIS is defined as a network model representation of the Sugeno-type fuzzy system employing the aptitudes of the adaptive network to eliminate the main problem of membership functions in the procedure of the fuzzy controller [22]. The Sugeno-type fuzzy model is defined as a mathematical type of fuzzy interface model explaining the linear input-output relationship defined in the following form using two inputs [36]:

(1) $$\begin{array}{c} \text{If}\;x\;\text{is}\;A\;\text{and}\;y\;\text{is}\;B\;\text{then}\;f=\left(x,y\right), \end{array}$$

where  *A* and *B* are the fuzzy sets shown in Figure 1 and the consequent *f*(*x*, *y*) is the crisp function of the *x* and *y* inputs; {*p*_*i*_, *q*_*i*_, *r*_*i*_} are parameter set provided from the membership functions equations in Sugeno fuzzy type. The zero order Sugeno FIS, a constant *f*(*x*, *y*), provides a smooth function of the input variables by adequate overlapping of the neighboring MFs to adjust the smoothness of the consequent crisp function by running the MFs through the ANFIS.

The membership functions are defined in the fuzzy sets to take into account the normality and convexity properties through a mathematical expression of the FIS. Based on the object of interest, the MFs could be linear, quadratic, or exponential in nature. In the learning phase of ANFIS method, a hybrid learning rule is employed. The hybrid learning rule is a combination of the Least-Squares Estimate (LSE) method aimed at calculating the error measurement and the backpropagation gradient descent for propagating from the output end toward the input end and using the error rate to update the parameters [22].

### 2.1. Architecture of the Model

**Figure 2:**
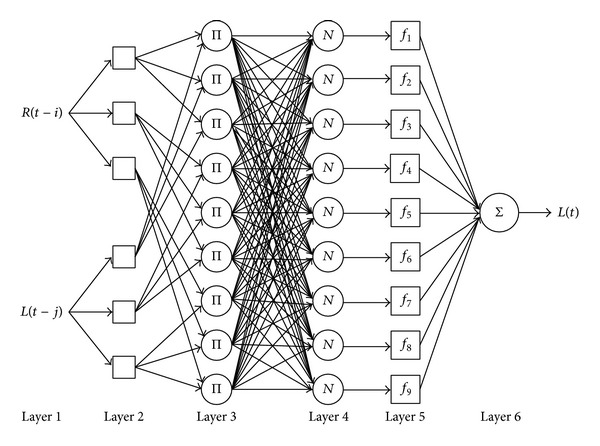
ANFIS architecture structure for forecasting the dam level.

The objective of this study is determining the performance of the new model to forecast the level of water in two different kinds of water resources, reservoirs and river basins, using the ANFIS model as a feed-forward network held neural network learning algorithm and fuzzy system with different types of MFs for each type of input. The designed ANFIS architecture includes two inputs, the daily rainfall at time (*t* − *i*) and water level at time (*t* − *j*), and one output, the water level at time *t*, shown in Figure 2, on the basis of the different time lags (*i*, *j*) considered in inputs. The square nodes demonstrate adaptive nodes representing the adjustable parameter sets, and the circle nodes are fixed ones.

The description of the structure of the noticed ANFIS model, including six layers, is shown as follows.

*Layer 1*. In this layer, the circle nodes are the input nodes that transfer the rainfall and the water level data as the inputs to the next layer. Based on the previous study done by Valizadeh et al. [37] on the forecasting the reservoir level using ANFIS used different time lag to determine the best model for the reservoir using solely two type of data, rainfall (*R*) and the level of reservoir (*L*); the set of inputs, [*R*(*t* − 1), *L*(*t* − 1)], notated here as *Rt*1*Lt*1, is used as the most evaluated pairs of sets, where *R* and *L* represent the rainfall and the water level data [37]. 

*Layer 2*. The square nodes in the second layer play as an MF in which the output is the membership grade of the given input variable, defined as follows:

(2) $$\begin{array}{c} O_{i}^{1}=\mu_{A_{i}}\left(R\right)\;\text{for}\;i=1,2,3,\\ O_{i}^{2}=\mu_{B_{i-3}}\left(L\right)\;\text{for}\;i=4,5,6, \end{array}$$

where *R* and *L* are input variables symbolizing the rainfall and water level, with *A*_*i*_ and *B*_*i*−3_ describing the linguistic fuzzy sets (High, Medium, and Low) in different ranges and shapes of MFs for each type of input. The ordinary procedure of the second layer of ANFIS in hydrological models uses the Gaussian or generalized bell shape for all inputs due to the smoothness and popularity of this distribution; however, depending on such data characteristics as the range of numbers, dispersion, and condensation, different MFs could be defined in ANFIS for each type of input. Notice that the range of fuzzy rules is specified automatically by program code on the basis of the range of the data sets and the number of sets for each input (High, Medium, and Low).

In the ANFIS model with the same types of MFs for the inputs, the generalized bell-shaped MF produced the most accurate result and is denoted as model GblGbl. On the basis of the testing combination of the MFs in ANFIS modeling, the most precise ANFIS model with different types of MFs considered in the study was GblGaus, consisting of the combination of a generalized bell-shaped MF for the input data representing daily rainfall and the Gaussian MF for the previous water level data in the case studies. The characteristics and details of the MFs are described as follows.

**Figure 3:**
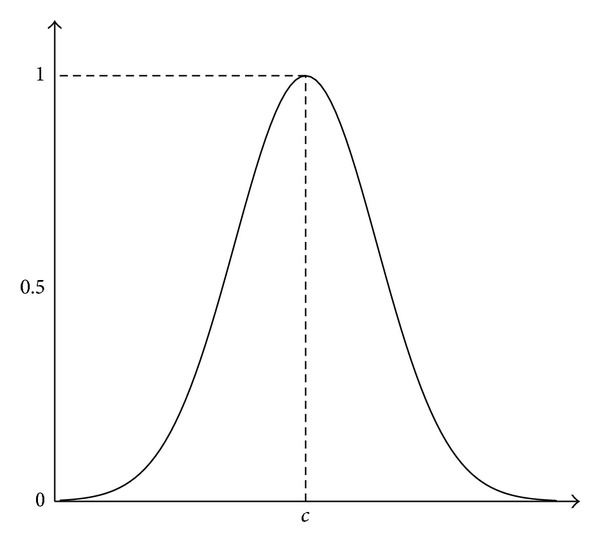
Shape of a Gaussian MF.

A* Gaussian MF* is classified as a probability distribution function (PDF) that creates a smooth boundary transition depending on the function parameters *σ* and *c* in the Gaussian MF formula, where *c* is the center of the MF and *σ* is a constant related to the width of the function, shown in Figure 3:

(3) $$\begin{array}{c} \text{gaussmf}\left(x;\sigma ,c\right)=e^{-{\left(\left(x-c\right)/\sigma \right)}^{2}}. \end{array}$$

**Figure 4:**
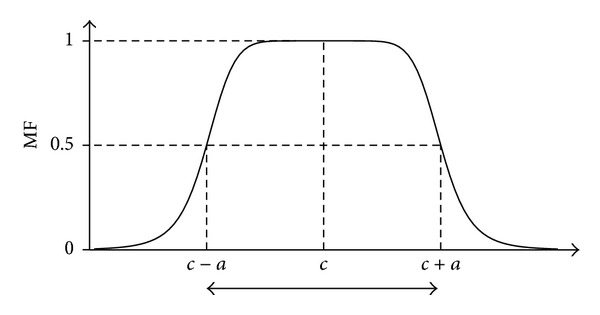
Shape of generalized bell-shaped MF.

**Figure 5:**
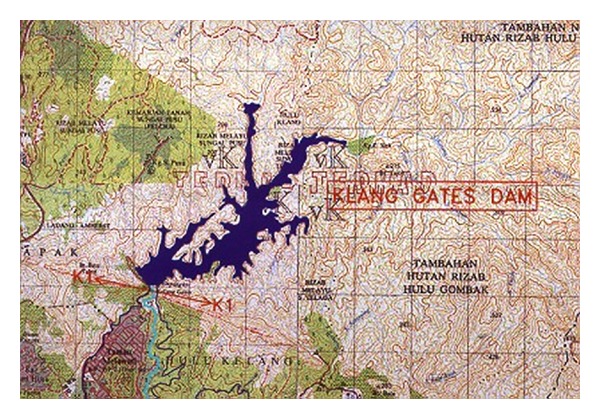
Klang Gate Dam Map.

The* Generalized Bell-Shaped MF* is a generalization of the Cauchy distribution employed in probability theory and is specified by three parameters {*a*, *b*, *c*}:

(4) $$\begin{array}{c} \text{gebelmf}\left(x;a,b,c\right)=\;\frac{1}{1+{\left|\left(x-c\right)/a\right|}^{2b}}, \end{array}$$

where the parameter *c* represents the center of the curve. Notice that the parameter *b* is almost positive. The advantage of the G-Bell MF is that it is possible to adjust the width of the curve by changing the *a* and *c* parameters and to control the slope by changing the parameter *b*, as shown in Figure 4 [38].

*Layer 3.* The output of this layer, firing strength, results from multiplying the two MFs obtained in the previous layer using an AND operator:

(5) $$\begin{array}{c} O_{k}^{3}=w_{k}=\mu_{A_{i}}\left(R\right)\times \mu_{B_{j}}\left(L\right)\;k=1,\ldots ,9\;\;i=j=1,2,3. \end{array}$$

*Layer 4.* The purpose of the fourth layer, named normalize firing strength, is to calculate the weight of the *i*th rule fringe strength:

(6) $$\begin{array}{c} O_{k}^{4}=\bar{w_{i}}=\frac{w_{i}}{\sum w_{i}}\;i=1,\ldots ,9. \end{array}$$

*Layer 5.* This layer corresponds to the consequent nodes that compute the contribution of each *i*th rule aimed at the model output:

(7) $$\begin{array}{c} O_{k}^{5}=\bar{w_{i}}f_{i}=\bar{w_{i}}\left(p_{i}R+q_{i}L+r_{i}\right), \end{array}$$

where *w*_*i*_ is the normal firing strength and the parameter set {*p*_*i*_, *q*_*i*_, *r*_*i*_} exists from the Sugeno fuzzy model [36].

*Layer 6.* Output node: this sole circle node computes the overall output by summing all previous layer signals:

(8) $$\begin{array}{c} O_{k}^{6}=\text{LEVEL}=\overset{2}{\underset{i=1}{\sum }}\bar{w_{i}}f_{i}=\frac{\sum_{i=1}^{2}w_{i}f_{i}}{\sum_{i=1}^{2}w_{i}}. \end{array}$$

### 2.2. Study of Area and Model Development

The reservoir of the Klang Gates Dam is located on the west coast of peninsular Malaysia in Taman Melawati and is influenced by Kuala Lumpur, Klang, Selangor state, Gombak, Hulu Langat. The dam location is at latitude 3 13′ 58′′ N and longitude 101 45′ 0′′ E. The dimensions of the reservoir dam are 138.7 m in length and 36.89 m in height, providing a 25.1e6 m^3^ reserved water capacity operated for flood control, hydropower generation, and industrial and domestic water supply. The Klang Gates Dam is formed by joining the 11 main tributaries that make up the Klang River and has a 1290 km^2^ basin area and a 120 km total length. The upper section of the Klang catchment is covered by forest, although the lower section consists mostly of a developed urban area, which is the major source of sediment loads and flood peaks.

**Figure 6:**
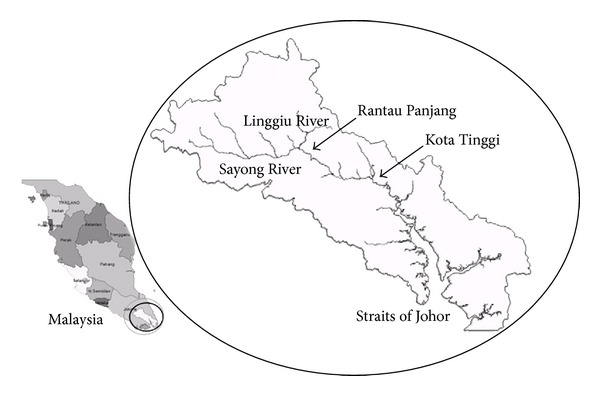
Johor River Basin Map.

The Rantau Panjang, Johor is located around 42 kilometers north east of Johor Bahru, Malaysia. Rantau Panjang station that is on the upper stream of the Kota Tinggi has been selected as a case study area located along the banks of Johor River. Normal water level at Rantau Panjang is 4 meters while at Kota Tinggi it is 1 meter. The Johor River is 122.7 km in length and drains an area of 2,636 km^2^ (Figure 6). The main tributaries of the river are Sayong River and Linggiu River. The river flow originates from Mount Gemuruh (109 m) and discharges the flow into the Straits of Johor. The mouth of the river is 0 m. Annual average precipitation for the Johor River catchment is 2,470 mm.

**Figure 7:**
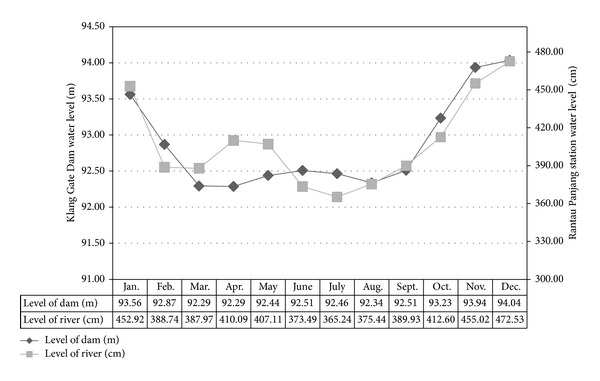
Monthly average water level at the Klang Dam and Rantau Panjang.

For this study, data observations of the daily rainfall and Klang Dam level from 1997 to 2008 and Rantau Panjang station water level and daily rainfall between 1963 and 2008 are employed.  Figure 7 shows the average of water level at the two introduced case studies describing that during May and August, the water level were fluctuated in their minimum levels; whereas, the level of the case studies have been increased from August and reached to the peak on January and December showing the significance of the operation to prevent the flood during these months.

According to the development of the ANFIS model, the observation data are divided into training, checking, and testing categories with the grouping ratio of 8 : 2 : 2, respectively [39]. In order to calibrate the ANFIS model, different number of fuzzy sets and also types of membership functions are examined to find the optimal model. Moreover, the most desirable step size to provide smooth tuning in both models is obtained by examining the most possible set of the step size resulted, [0.8, 0.9, 1.00], which represents the step size, the step size decrease rate, and the step size increase rate, respectively.

In order to evaluate the performance of the ANFIS models, the following four statistical criteria are considered: (1) the correlation coefficient (*R*) defines the weight of the relationship between the observed and forecasted dam levels, (2) the root mean square error (RMSE) calculates the residual value between the actual and forecasted dam levels, (3) the mean absolute percentage error (MAPE) evaluates the fitness of the time series data specifically for trending procedures, and (4) the mean absolute error (MAE):

(9) $$\begin{array}{c} R=\frac{\sum_{i=1}^{n}\left(Q_{i}^{O}-{\overset{-}{Q}}^{O}\right)\left(Q_{i}^{F}-{\overset{-}{Q}}^{F}\right)}{\sqrt{\sum_{i=1}^{n}{\left(Q_{i}^{O}-{\overset{-}{Q}}^{O}\right)}^{2}{\left(Q_{i}^{F}-{\overset{-}{Q}}^{F}\right)}^{2}}\;},\\ \text{RMSE = }{\left[\overset{n}{\underset{i=1}{\sum }}\frac{{\left(Q_{i}^{O}-Q_{i}^{F}\right)}^{2}}{n}\right]}^{0.5},\\ \text{MAPE =}\frac{1}{n}\overset{n}{\underset{i=1}{\sum }}\left|\frac{Q_{i}^{F}-Q_{i}^{O}}{Q_{i}^{O}}\right|,\\ \text{MAE =}\overset{n}{\underset{i=1}{\sum }}\frac{\left|Q_{i}^{O}-Q_{i}^{F}\right|}{n}, \end{array}$$

where *Q* ^*O*^ and *Q* ^*F*^ are the actual and forecasted dam levels, respectively, and $\overset{-}{Q}$ represents the average reservoir level. Values of the correlation coefficient that are closer to unity illustrate a stronger linear relationship between the actual and forecasted values and a perfect fit for the observed and forecasted values is indicated when the MAPE becomes zero.

## 3. Results and Discussion

**Figure 8:**
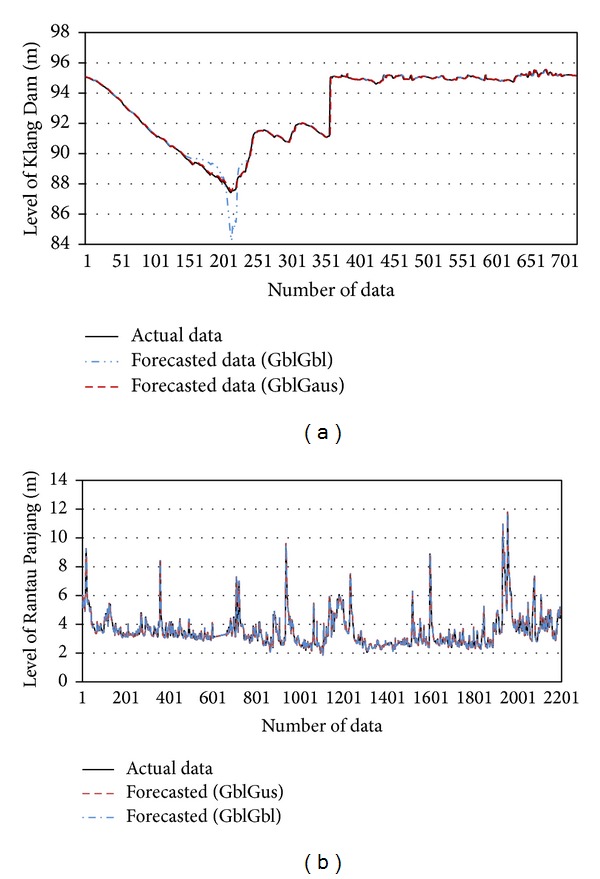
Actual and forecasting time series data.

On the basis of the basic ANFIS structure model, the level of water is forecasted by the traditional and the new fuzzification pattern defined in methodology. Since the model employs the minimum type of inputs needed the one time lag is considered for the inputs that provided the most accurate result in previous study [37]. The comparison between the actual data and results of the two forecasted models for the two case studies are shown in Figure 8. The model that uses a generalized bell-shaped MF for the rainfall input data and a Gaussian MF for the water level input data provides a better fit between the actual and forecasted data in both types of water resources compared with the model that uses a generalized bell-shaped MF for the two input data sets and gives unmatched estimation in Klang Gate Dam, when the water level reached minimum, because of the model problem in tuning itself in the range of water level. Moreover, the estimation of water level in Rantau Panjang station demonstrate more stress in forecasting level of stream using the generalized bell-shaped MF for the two input data sets.

**Figure 9:**
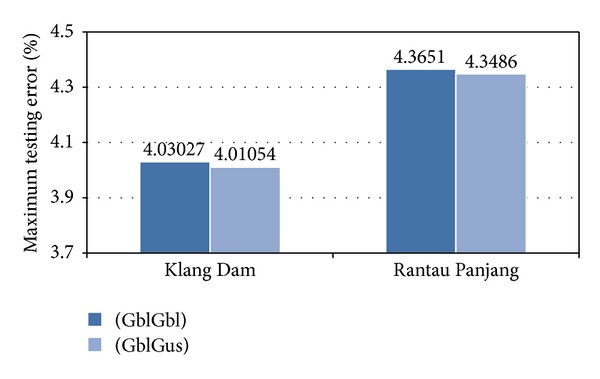
Maximum testing error (%).

**Figure 10:**
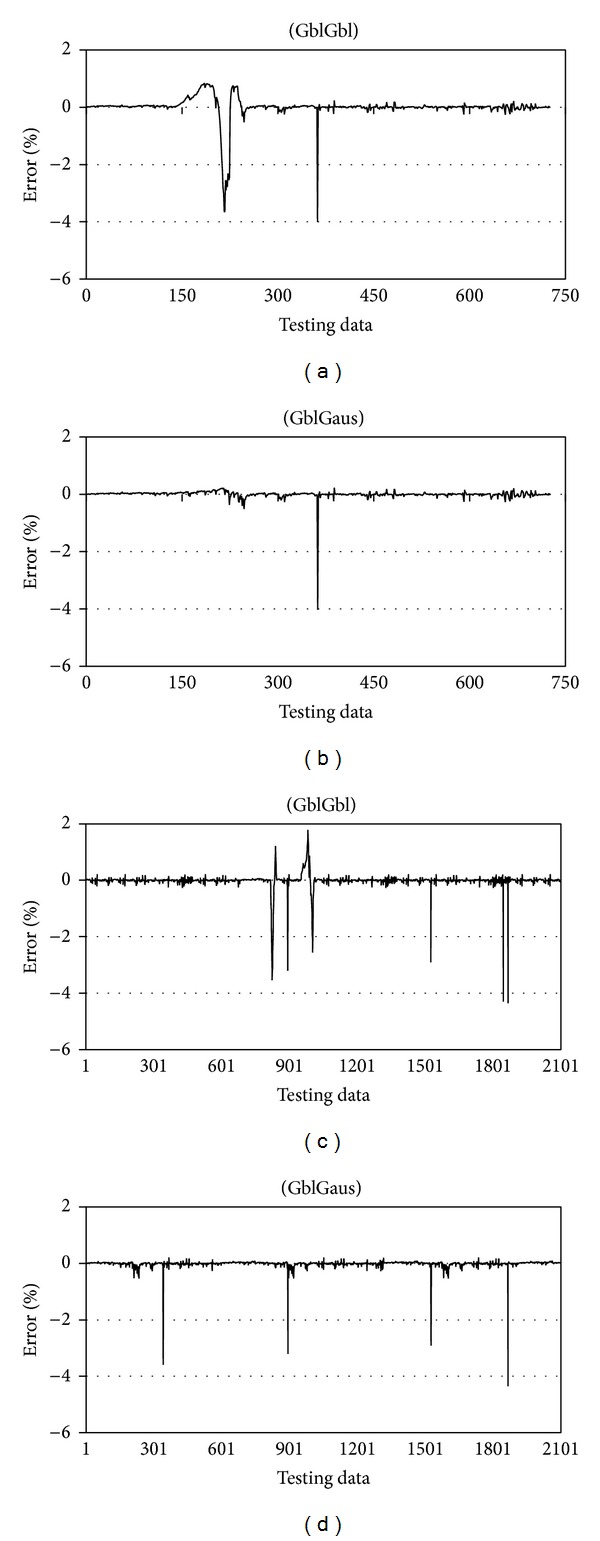
Error performance.

In spite of the Figure 9 showing the performance of the two introduced model are almost same, the number of sudden changes in maximum errors of the GblGaus model illustrated in Figure 10 are fewer and the errors are in lower degree rather than the traditional model, GblGbl. Moreover, notice that the model employs different MFs for the inputs not only have less stress compared with the traditional ANFIS model, but also the weakness of the traditional model in low water levels has been solved using developed fuzzifying method in ANFIS.

**Figure 11:**
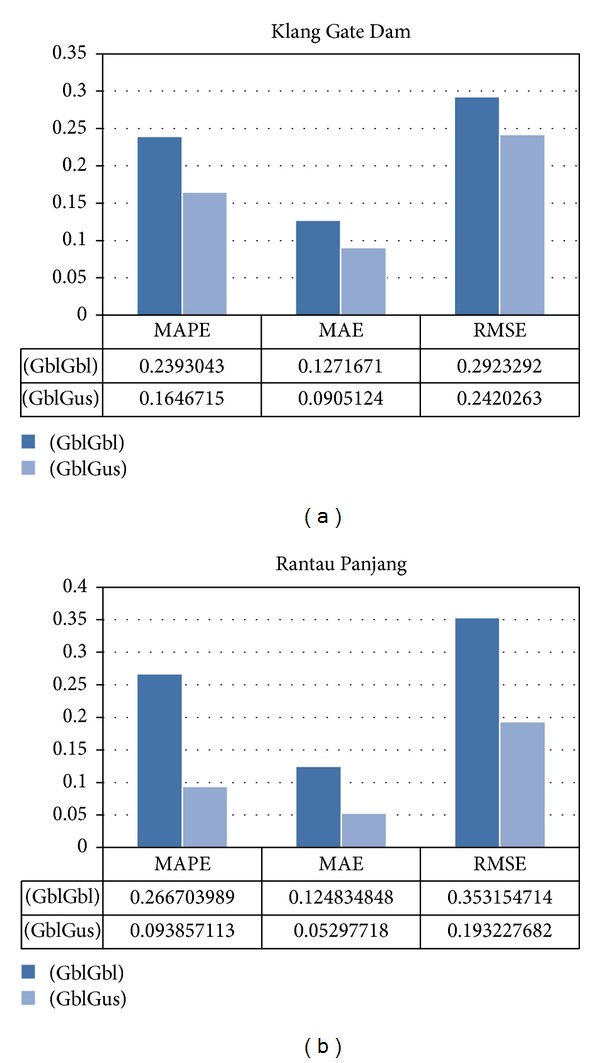
Model statistics of case studies.

**Figure 12:**
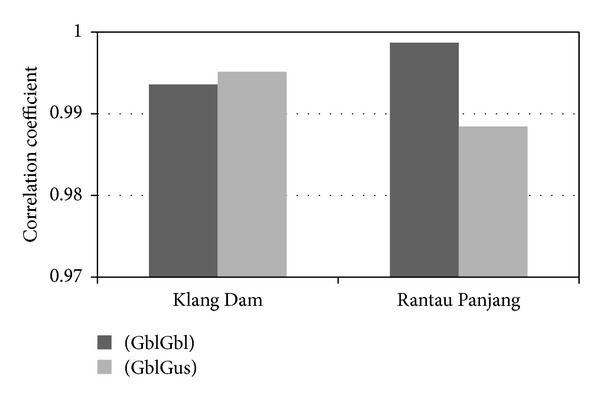
Correlation coefficient.

According to the three statistical evaluations considered in Figure 11, the model that utilized different types of MFs achieved much better results; especially the difference of the coefficient numbers is greater in Rantau Panjang station than the Klang Gate Dam. Specifically, the root mean square error in the GblGaus model was over 0.05 and 0.15 smaller than that in the GblGbl model in Klang Gate Dam and in Rantau Panjang station, respectively, demonstrating the superior accuracy of the GblGaus model. The significantly smaller MAPE value of the GblGaus model shows the higher accuracy of the data series. Another statistical coefficient showing the relationship between the actual and estimated data is the correlation coefficient (*R*), as illustrated in Figure 12. Instead of the difference between the correlation coefficients of the two models of Klang Dam which were approximately the same, in Rantau Panjang station the model using a generalized bell-shaped MF and a Gaussian MF indicates that the linear relationship between the actual and forecasted data is considerably improved compared to the model using two generalized bell-shaped MF inputs.

## 4. Summary and Conclusions

Forecasting of the water level in two different types of case studies showing the characteristics of reservoirs and stream flow is evaluated using two different ANFIS models to examine the performance of the new fuzzifying pattern in ANFIS model to identify the precision of the ANFIS model using different types of MFs in contrast to that of the regular ANFIS model using the same type of MFs for all inputs in different water resources. Due to the smoothness and popularity of the generalized bell-shaped MF and the Gaussian MF, this study used a combination of these MFs for the models. Regarding the results of the model using the generalized bell-shaped MF for the rainfall input and the Gaussian MF for the previous water level produced the most accurate results in two case studies compared with the model that employed the generalized bell-shaped MF for the two types of inputs. The statistical criteria also show a better fit and a stronger linear relationship between the actual and forecasted data in the model using two different types of MFs for the two types of inputs. Finally, this study proved the ability of the new model utilizing the different types of membership functions in the ANFIS models in different type of water resources and hydrological conditions helping water resource management yield results with greater precision in ANFIS model. Future studies will focus on mathematical proof of the fuzzy section of the model with different combinations of MFs, comparing the new method with the other AI models and utilizing the method in other different hydrological estimation to support the results of this study.
